# VX-765 Alleviates β-Amyloid Deposition and Secondary Degeneration in the Ipsilateral Hippocampus and Ameliorates Cognitive Decline after Focal Cortical Infarction in Rats

**DOI:** 10.1007/s12031-022-02088-6

**Published:** 2022-11-28

**Authors:** Dawei Dong, Aihui Ren, Ying Yang, Jiayi Su, Libin Liu, Wenyan Zhuo, Yubin Liang

**Affiliations:** 1 Department of Neurology and Stroke Center, The First Affiliated Hospital, Jinan University, Guangzhou, Guangdong China; 2 Department of Neurology, Zhuhai People’s Hospital (Zhuhai Hospital Affiliated With Jinan University), Zhuhai, China

**Keywords:** Cerebral infarction, Secondary degeneration, Hippocampus, Pyroptosis, VX-765

## Abstract

Focal cortical infarction leads to secondary degeneration of the ipsilateral hippocampus, which is associated with poststroke cognitive impairment. VX-765 is a potent small-molecule caspase-1 inhibitor that protects against central nervous system diseases. The present study aimed to determine the protective effects of VX-765 on β-amyloid (Aβ) deposition and secondary degeneration in the hippocampus as well as cognitive decline after cortical infarction. Sprague–Dawley rats were used to establish a distal middle cerebral artery occlusion (dMCAO) model and randomly divided into the vehicle and VX-765 groups. Rats in the vehicle and VX-765 groups, respectively, were subcutaneously injected with VX-765 (50 mg/kg/d) and an isopycnic vehicle once a day for 28 days, starting 1 h after dMCAO. At the end of this 28-day period, cognitive impairment was evaluated with the Morris water maze, and secondary hippocampal damage was evaluated with Nissl staining and immunostaining methods. Neuronal damage and pyroptosis were detected by TUNEL and immunoblotting. The results revealed that VX-765 treatment ameliorated poststroke cognitive dysfunction after ischemia. VX-765 reduced Aβ deposition, neuronal loss, and glial activation compared with the vehicle control. In addition, VX-765 treatment increased BDNF levels and normalized synaptophysin protein levels in the hippocampus after cortical infarction. Notably, VX-765 treatment significantly reduced the expression of the pyroptosis-related molecules caspase-1, NLRP3, apoptosis-associated speck-like protein (ASC), gasdermin D, IL-1β, and IL-18. Additionally, VX-765 significantly decreased the numbers of TUNEL-positive cells and the levels of Bax and cleaved caspase-3 (cC3) and enhanced the levels of Bcl-2 and Bcl-xl after ischemia. Inflammatory pathways, such as the NF-κB and mitogen-activated protein kinase (MAPK) pathways, were inhibited by VX-765 treatment after ischemia. These findings revealed that VX-765 reduced Aβ deposition, pyroptosis, and apoptosis in the ipsilateral hippocampus, which may be associated with reduced secondary degeneration and cognitive decline following focal cortical infarction.

## Introduction

Focal cortical infarction after distal middle cerebral artery occlusion (dMCAO) not only results in primary cerebral ischemia but also causes secondary degeneration in regions that have synaptic connections with infarct-like lesions (Zhang et al. 2011). A previous study found that neuronal injury and glial hyperplasia occurred in the CA1 region of the ipsilateral hippocampus of dMCAO rats within 7 days. However, these injuries were not caused by ischemia in the hippocampus (Uchida et al. 2010). The most common pathological changes involved in secondary degeneration include neuronal loss, glial degeneration, axonal degeneration, β-amyloid (Aβ) deposition, and hyperphosphorylation of Tau protein (Groen et al. 2005; Dong et al. 2014). These can lead to neuronal damage and even sustained dementia (Tamura et al. 1991; Freret et al. 2006). Therefore, there is a need to explore approaches for protecting against secondary neurodegeneration after ischemia.

Pyroptosis is a form of inflammatory programmed cell death that is triggered by NLRP3 inflammasome activation by caspase-1. Caspase-1 can induce pyroptosis through a mechanism that is dependent on the expression of the pyroptosis mediator gasdermin D (GSDMD). It is distinct from other forms of cell death in the sense that it is associated with the activation of inflammasomes mediated by GSDMD (Miao et al. 2010; McKenzie et al. 2020). Several studies have reported that pyroptosis plays a critical role in the pathogenesis of neurodegenerative diseases such as Alzheimer’s disease (AD) (Tan et al. 2014; Olsen and Singhrao 2016). β-Amyloid peptide-induced caspase-1 activation has been reported to inhibit autophagy in the cortex and hippocampus causing neurodegeneration and memory loss in an AD model (Alvarez-Arellano et al. 2018). Caspase-1 inhibition with the compound VX-765 improves episodic and spatial memory deficits and neuroinflammation in the J20 AD mouse model (Flores et al. 2018). These findings show that caspase-1 inhibition after focal cortical infarction reduces the detrimental outcomes of secondary degeneration.

Active caspase-1 induces pyroptosis, a necrotic form of regulated cell death, which promotes the release of intracellular proinflammatory molecules, including IL-1 and IL-18 (Miao et al. 2010). VX-765 is a potent and selective small-molecule caspase-1 inhibitor with the ability to cross the blood–brain barrier (Boxer et al. 2010). VX-765 has been shown to inhibit inflammatory diseases in the central nervous system. A recent study reported that VX-765 can reduce the deposition of Aβ peptide, mitigate brain inflammation, and improve hippocampal synaptophysin levels in AD mice (Flores et al. 2018). In addition, VX-765 prevents MCAO-induced brain damage and reduces microglia-mediated neuroinflammation by inhibiting microglial activation (Li et al. 2019). However, the potential effects of VX-765 on secondary degeneration in the nonischemic hippocampus and cognitive decline after ischemic stroke are unclear.

This study aimed to determine whether VX-765 can prevent cognitive decline and secondary degeneration in the ipsilateral hippocampus after focal cerebral infarction and to explore whether it exerts a neuroprotective effect by inhibiting inflammatory and pyroptotic pathways.

## Materials and Methods

### Study Design

All animal studies were conducted according to the guidelines of the Animal Care and Use Committee of Jinan University. Sprague–Dawley rats aged 12 months and weighing between 350 and 500 g were supplied by the Guangdong Medical Laboratory Animal Center. The rats were anesthetized using 10% chloral hydrate (3 ml/kg, BBI, China) (i.p.) solution, and a distal middle cerebral artery occlusion (dMCAO) model was established as previously described (Bederson et al. 1986). Sham-operated rats underwent the same surgical procedures except dMCAO electrocoagulation. At 1 h after dMCAO, the caspase-1 inhibitor VX-765 (50 mg/kg, MedChem Express, USA) was intraperitoneally injected once daily for 28 consecutive days after dMCAO as previously described (Flores et al. 2018). SD rats were randomly assigned to 3 groups: (1) the sham group, (2) the MCAO + vehicle group, and (3) the MCAO + VX-765 therapy group. Cognitive and learning function was determined 24 d after dMCAO in the Morris water maze test.

### Morris Water Maze

The Morris water maze (MWM) was used to evaluate spatial learning and memory in rats as previously described (Huang et al. 2018). Rats were subjected to the Morris water maze task for 5 d to evaluate their learning and memory abilities. Short-term and long-term memory abilities were assessed using probe trials at 24 and 72 h after the 5-day spatial learning period. The platform was removed, and the rats were placed in the quadrant opposite from the previous platform location. An HVS system was used to record the times the rats stayed on the platform and the number of times the rats crossed the former location of the platform during the probe trials. The HVS water maze program (WaterMaze 3, Evanston, IL) was used to record all the trial data.

### Tissue Preparation

Rats were deeply anesthetized using 10% chloral hydrate (3 ml/kg, i.p.). Brains were excised from six rats in each group and perfused with 100 ml of saline and 300 ml of 4% paraformaldehyde. The brain tissue was placed in 4% paraformaldehyde at 4 °C for 3 d. Then, the brain tissue was dehydrated using 30% sucrose and embedded in OCT (SAKURA, USA). A 10-μm-thick coronal brain section was cut using a frozen microtome (Leica Biosystems, Germany). The hippocampal tissue on the same side as the infarction was excised from the other six rats in each group. Hippocampal tissues were immediately was quickly frozen and stored at – 80 °C until they were used for protein extraction.

### Nissl Staining

Frozen brain sections were washed three times for 5 min each in 0.01 M PBS and incubated with Nissl reagent A (Wanleibio, China) at room temperature (RT) for 30 min. Brain sections were washed with distilled water for 10 s. Sections were incubated with Nissl reagent B until the Nissl bodies were clearly visible under the microscope.

#### Immunofluorescence

Frozen sections were blocked with 5% goat serum for 1 h and incubated at 4 °C with diluted primary antibodies against NeuN (1: 300, cat. # ab104224, Abcam), GFAP (1: 300, cat. # ab7260, Abcam), Iba-1 (1: 300, cat. # ab178846, Abcam), caspase-1 (1: 100, cat. # sc-56036, Santa Cruz), and Aβ (1: 300, cat. # SIG-39151, Covance) for 24 h. The sections were incubated for 1 h with the corresponding fluorescent secondary antibodies IgG H&L 488 or 594 (all diluted to 1: 200, Abcam, USA) at RT. The brain sections were counterstained with DAPI (Beyotime, China) for 2 min, sealed with a fluorescence-quencher agent (CST, USA), and photographed under a fluorescence microscope (Leica DM1000, Germany).

### Western Blotting

Western blotting was performed as previously described (Huang et al. 2018) with the diluted primary antibodies NeuN (1: 300, cat. # ab104224, Abcam), GFAP (1: 300, cat. # ab7260, Abcam), Iba-1 (1: 300, cat. # ab178846, Abcam), caspase-1 (1: 100, cat. # sc-56036, Santa Cruz), NLRP3 (1: 1,000, cat. # DF7438, Affinity), ASC (1: 1,000, cat. # ab47092, Abcam), Gasdermin D (GSDMD, 1: 500, cat. # sc-393581, Santa Cruz), IL-1β (1: 1,000, cat. # ab254360, Abcam), IL-18 (1: 1000, cat. # ab207323, Abcam), BDNF (1: 1000, cat. # ab108319, Abcam), PSD95 (1: 1000, cat. # ab238135, Abcam), Syna (1: 1000, cat. # ab32127, Abcam), and GAP43 (1: 1000, cat. # ab75810, Abcam) and incubated for 12 h at 4 °C. After being washed three times with 0.1 M PBS, the membranes were incubated with HRP-conjugated anti-rabbit or anti-mouse secondary antibodies (diluted to 1: 5000, Abcam, USA) for 2 h at RT. Finally, the protein bands were visualized using a Western blotting detection kit (Wanleibio, China).

### TdT-Mediated dUTP Nick-End Labeling (TUNEL)

The frozen brain slices were washed 3 times for 5 min each with 0.01 M PBS and 0.1%. TUNEL staining was performed using a fluorescein kit (Roche, Germany) following the manufacturer’s instructions. TUNEL-positive cells were observed under a fluorescence microscope.

### Statistical Analysis

Data are expressed as the mean ± SEM. All statistical analyses were performed using SPSS 13.0 (Chicago, USA). The data were analyzed using one-way analysis of variance followed by the Bonferroni test for multiple comparisons. *P* < 0.05 was considered to be statistically significant.

## Results

### VX-765 Improves Cognitive Impairment after dMCAO

**Fig. 1:**
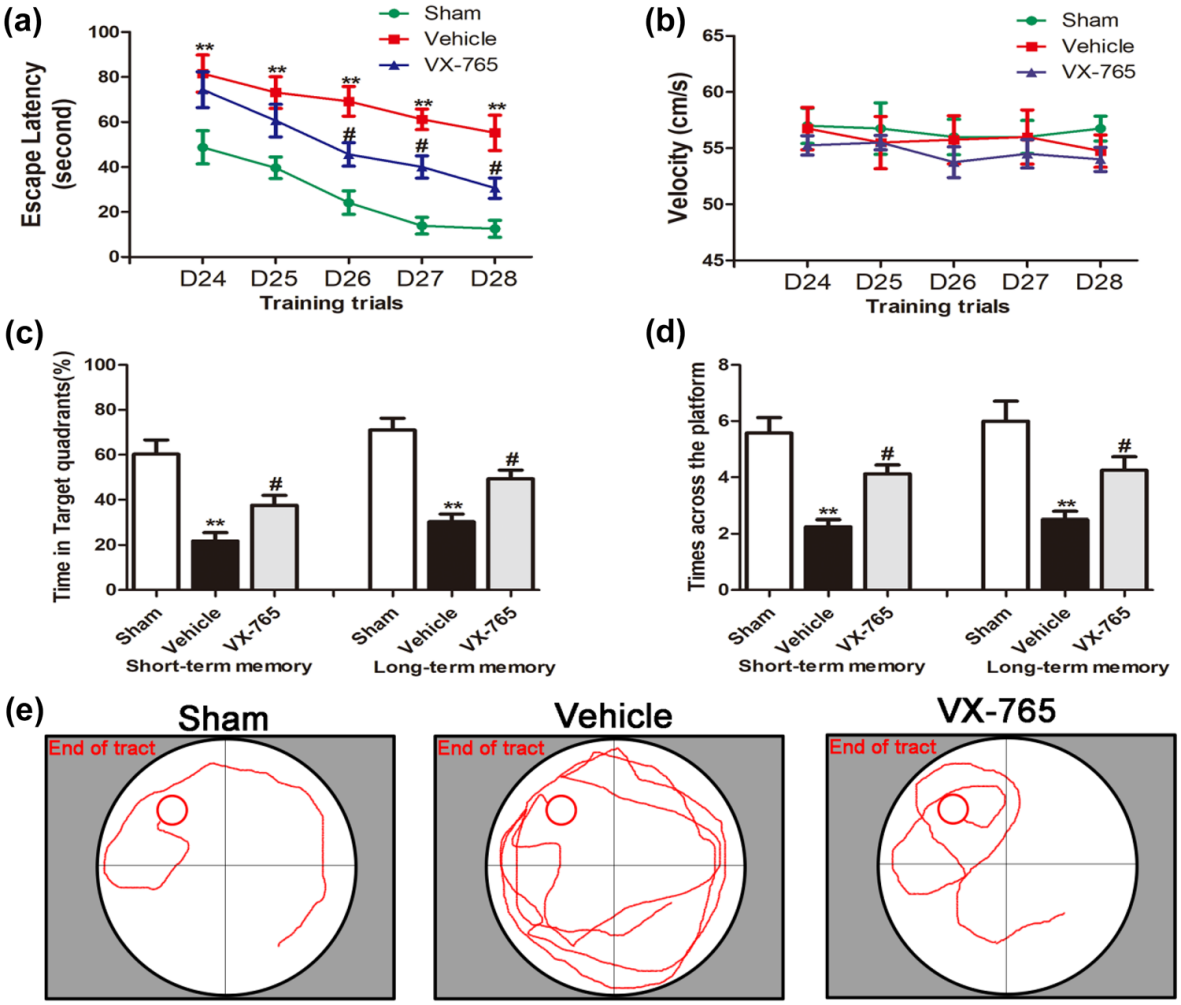
VX-765 improved cognitive decline after ischemia, as revealed by the Morris water maze test. **a** The latency of the rats to reach the platform was measured to assess the memory ability of the three groups after a 5-day training trial following ischemia. **b** No significant difference was observed in swimming speed among the 3 groups. **c** The percentage of time spent in the target quadrant for the 3 groups. **d** The numbers of platform crossings recorded in the 3 groups. **e** The swimming paths of the sham, vehicle, and VX-765 groups on the last day of the test. *n* = 12 per group. Data are shown as the mean ± SEM. ^*^P < 0.05 and ^**^P < 0.01 versus sham group; ^#^*P* < 0.05 versus vehicle group

Focal cortical infarction leads to secondary neuronal damage in the ipsilateral nonischemic hippocampus, which may result in cognitive disorders. Rats in the vehicle group took longer to reach the hidden platform and spent less time in the target quadrant than sham control rats. Additionally, rats in the vehicle group crossed the previous platform location fewer times than rats in the sham group (*P* < 0.05, Fig. 1). There was no significant difference in swimming speed between the two groups from Day 24 to Day 28. These MWM results revealed that cognitive decline occurred after ischemic stroke (Fig. 1b). Treatment with VX-765 reduced the latency time on Days 26, 27, and 28 following ischemic stroke during the training days (*P* < 0.05). A probe test was conducted after 5 days of training to assess short-term memory (after 24 h) and long-term memory (after 72 h) on Day 29 and Day 31, respectively. Compared with the sham group, the vehicle group displayed a longer path distance in all trials (*P* < 0.05). Rats in the VX-765 group spent a significantly longer time in the target quadrant and crossed the platform more times than rats in the vehicle group (*P* < 0.05, Fig. 1c, d). The movement paths were similar between the VX-765 treatment group and the sham group (Fig. 1e). These findings suggested that VX-765 treatment improved cognitive decline after focal cortical infarction in rats.

### VX-765 Alleviates Secondary Degeneration in the Ipsilateral Hippocampus Following dMCAO

**Fig. 2:**
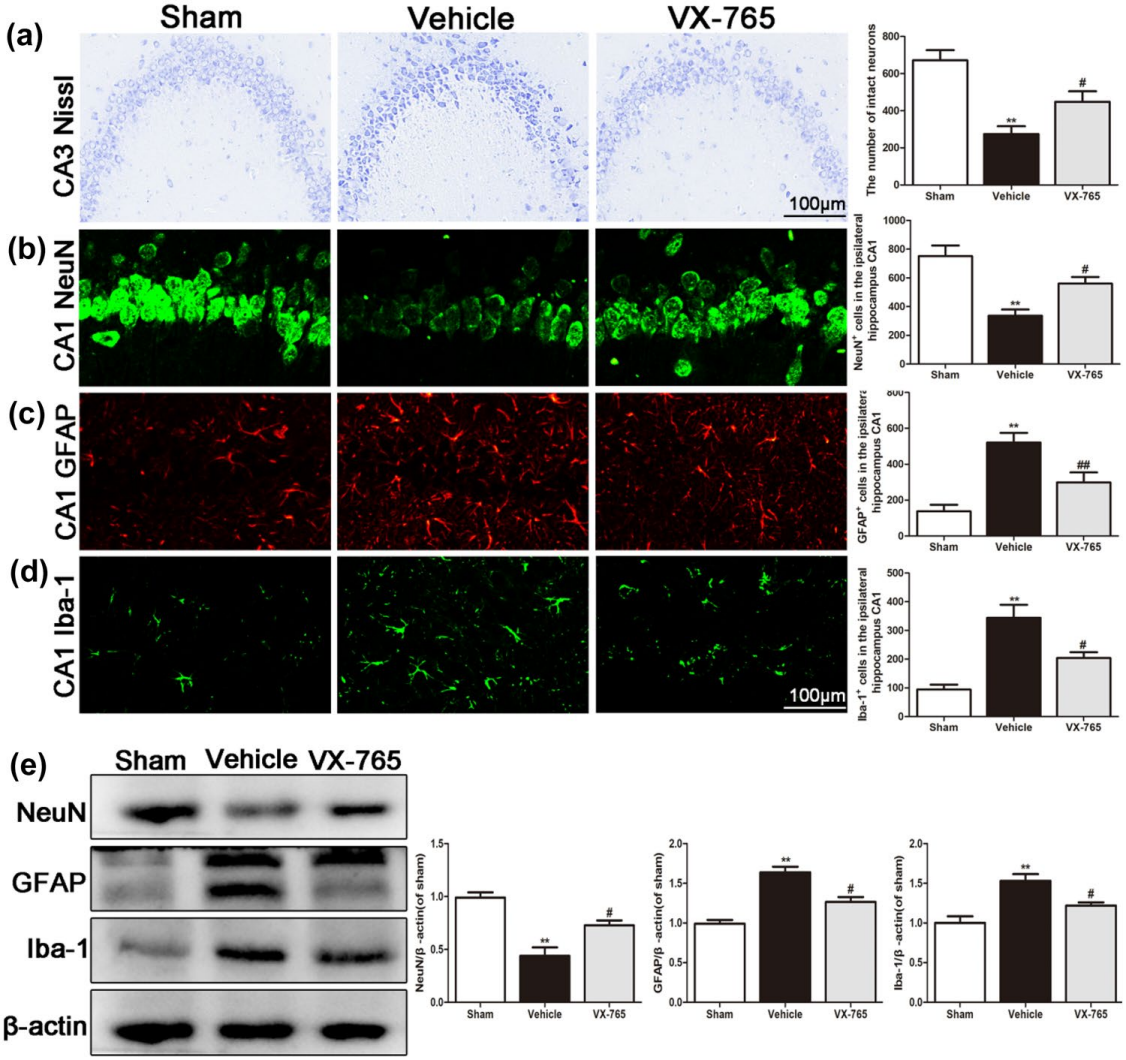
VX-765 attenuates secondary degeneration in the ipsilateral hippocampus at 28 days following dMCAO. **a** Nissl images showing intact neurons and immunofluorescent images of **b** NeuN, **c** GFAP, and **d** Iba-1 in the ipsilateral hippocampus after dMCAO. Scale bar: 100 μm. **e** Immunoblotting analysis of the expression of NeuN, GFAP, and Iba-1 in the ipsilateral hippocampus after dMCAO, *n* = 6 per group. Data are shown as the mean ± SEM. ^**^P < 0.01 versus the sham group; ^#^*P* < 0.05 and ^##^*P* < 0.01 versus the vehicle group

Focal cortical infarction causes neuronal apoptosis and glial activation within the ipsilateral hippocampus. Nissl staining revealed atrophic neurons with shrunken cytoplasm and abnormal nuclei in the vehicle group 28 days after cortical infarction in the ipsilateral hippocampus (Fig. 2a). The number of intact neurons in the ipsilateral hippocampus in the VX-765 group was significantly higher than that in the vehicle group at 28 days after dMCAO (*P* < 0.05). Congruent with the results of the Nissl staining assay, the number of NeuN^+^-positive cells in the ipsilateral hippocampus was markedly decreased in the vehicle and VX-765 groups at 28 days following dMCAO. VX-765 treatment significantly reversed the NeuN^+^ cell loss in the ipsilateral hippocampus after dMCAO compared with the vehicle treatment (*P* < 0.05, Fig. 2b). Conversely, the number of GFAP^+^-positive cells and Iba-1^+^-positive cells in the ipsilateral hippocampus was higher in the vehicle group than in the sham rats. However, at 28 days following dMCAO, the number of cells was significantly lower in the VX-765 group than in the vehicle group (*P* < 0.05, Fig. 2c and d). The immunoblotting results were consistent with those of the immunofluorescence assay (*P* < 0.05, Fig. 2e). These results showed that VX-765 alleviated secondary degeneration in the ipsilateral hippocampus following ischemia.

### VX-765 Reduces Aβ Deposition and Synapse Injury in the Ipsilateral Hippocampus after dMCAO

**Fig. 3:**
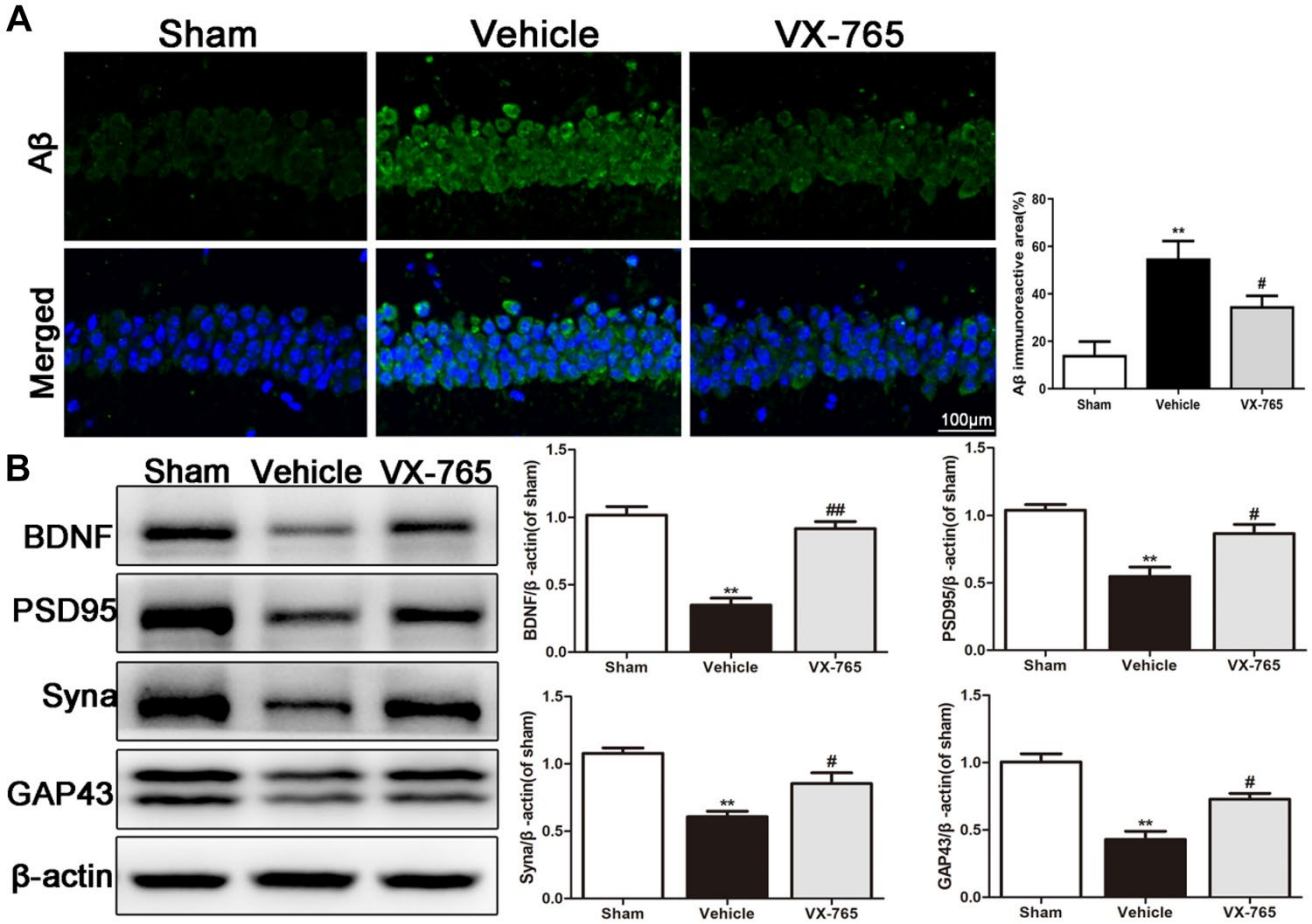
VX-765 decreases Aβ deposition and synaptic damage in the ipsilateral hippocampus 28 days following dMCAO. **a** Immunostaining showing Aβ accumulation in the ipsilateral hippocampus after dMCAO. Scale bar: 100 μm. **b** Immunoblotting images displaying BDNF, PSD95, Syna, and GAP43 levels in the ipsilateral hippocampus after dMCAO, *n* = 6 per group. Data are shown as the mean ± SEM. ^**^P < 0.01 versus the sham group; ^#^*P* < 0.05 and ^##^*P* < 0.01 versus the vehicle group

According to previous research, Aβ deposits can arise within the ipsilateral thalamic region following cortical infarction. Immunofluorescence results indicated that the increased Aβ levels in the hippocampus induced by dMCAO significantly decreased after VX-765 treatment (*P* < 0.05, Fig. 3a). Aβ accumulation can cause synaptic damage. BDNF, PSD95, GAP43, and Syna are neuronal plasticity markers that play vital roles in synapse reconstruction and recovery (Mardones et al. 2019). As expected, the expression of BDNF, PSD95, GAP43, and Syna in the vehicle group was lower than that in the sham group. However, VX-765 treatment significantly increased the expression of these synaptic proteins (*P* < 0.05, Fig. 3b). Therefore, VX-765 inhibited ischemia-induced Aβ accumulation and protected neurons from dMCAO-induced synaptic damage.

### VX-765 Inhibits Pyroptosis in the Ipsilateral Hippocampus after dMCAO

**Fig. 4:**
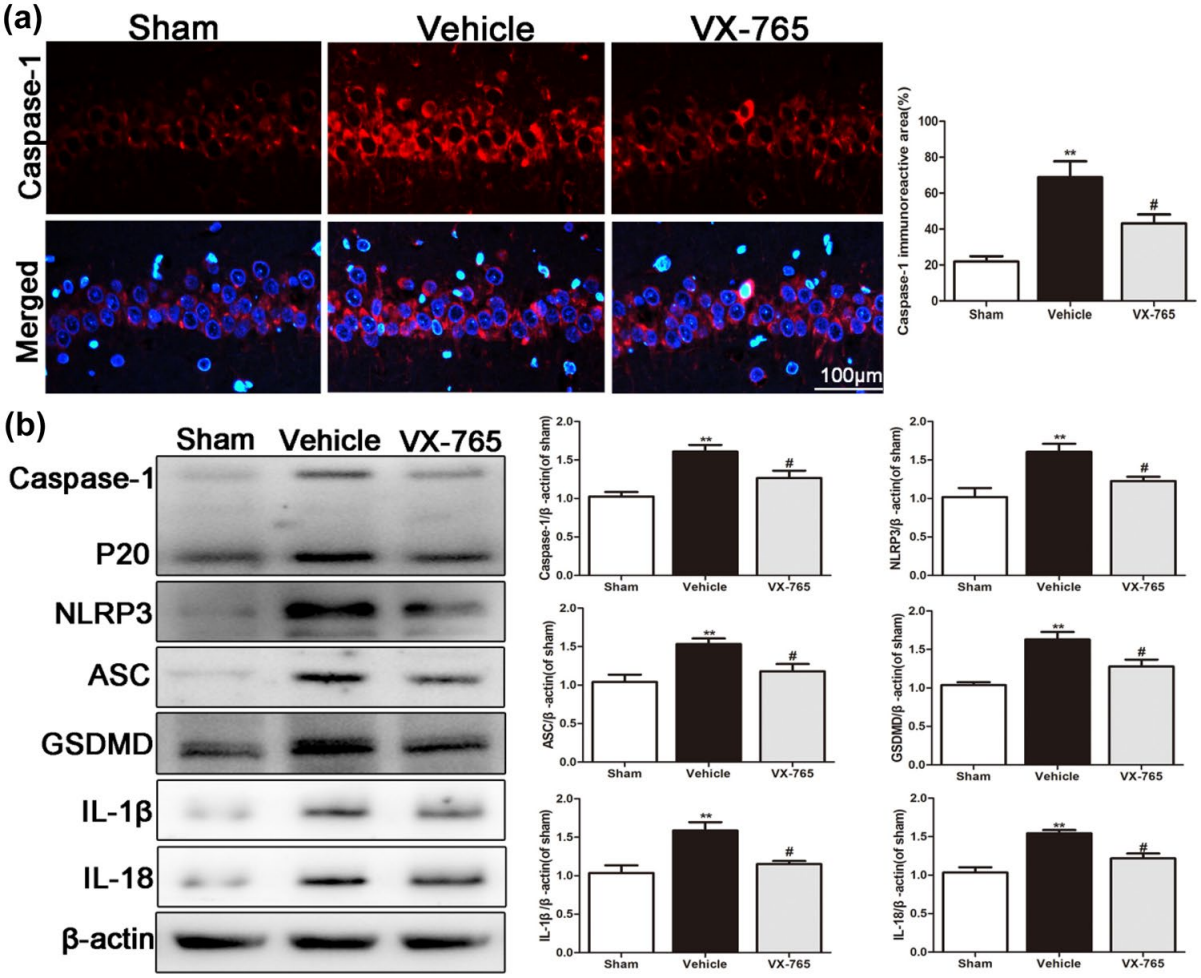
VX-765 inhibits pyroptosis in the ipsilateral hippocampus 28 days after dMCAO. **a** Representative immunofluorescent staining of caspase-1 in the ipsilateral hippocampus after dMCAO. Scale bar: 100 μm. **b** Western blot images indicating the expression of caspase-1, NLRP3, ASC, GSDMD, IL-1β, and IL-8 in the ipsilateral hippocampus after dMCAO, *n* = 6 per group. Data are shown as the mean ± SEM. ^**^P < 0.01 versus the sham group; ^#^*P* < 0.05 versus the vehicle group

Inhibition of caspase-1 ameliorates ipsilateral hippocampal dysfunction and integrity by suppressing pyroptosis activation. As shown in Fig. 4a, extensive caspase-1 immunoreactivity was observed in the vehicle group 28 days following ischemic stroke. However, VX-765 treatment significantly decreased caspase-1 staining in the ipsilateral hippocampal CA1 region (*P* < 0.05). The pyroptosis-related molecules caspase-1, NLRP3, GSDMD, ASC, IL-1β, and IL-18 were upregulated in the ipsilateral hippocampus after dMCAO but were reduced following treatment with VX-765 (*P* < 0.05, Fig. 4b). These results revealed that VX-765 treatment blocked inflammation and pyroptosis in the ipsilateral hippocampus after dMCAO.

### VX-765 Decreases TUNEL Staining and Apoptosis in the Ipsilateral Hippocampus after dMCAO

**Fig. 5:**
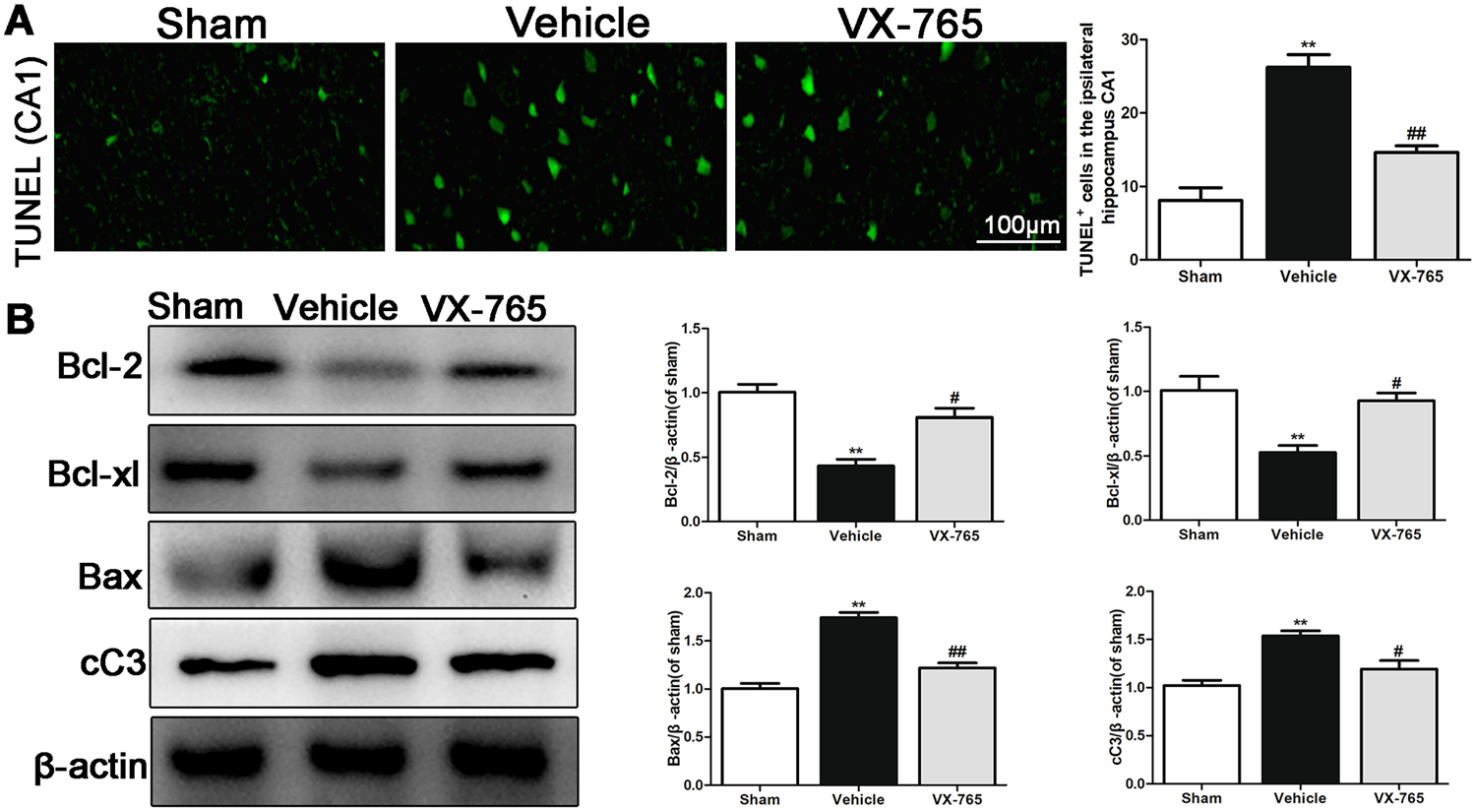
VX-765 reduces TUNEL positivity and apoptosis in the ipsilateral hippocampus 28 days following dMCAO. **a** TUNEL cells in the ipsilateral hippocampus following dMCAO. Scale bar: 100 μm. **b** Western blot images showing the expression of Bcl-2, Bcl-xl, Bax, and cC3 in the ipsilateral hippocampus following dMCAO, *n* = 6 per group. Data are shown as the mean ± SEM. ^**^P < 0.01 versus the sham group; ^#^*P* < 0.05 and ^##^*P* < 0.01 versus the vehicle group

Cells with positive TUNEL signal, which reflects late-stage apoptosis, were observed in the ipsilateral hippocampus at 28 days following dMCAO. Figure 5a shows the number of TUNEL cells observed in the ipsilateral hippocampus of the vehicle group and the VX-765-treated group at 28 days following dMCAO. However, VX-765 treatment significantly reduced the number of TUNEL cells in the ipsilateral hippocampus following dMCAO (*P* < 0.05). Compared with the sham group, the Bcl-2 and Bcl-xl levels in the vehicle group were significantly lower in the ipsilateral hippocampus. VX-765 treatment significantly increased the levels of Bcl-2 and Bcl-xl in the ipsilateral hippocampus at 28 days after cortical infarction (*P* < 0.05, Fig. 5b). However, the expression of Bax and cC3 (cleaved caspase-1) levels in the ipsilateral hippocampus was significantly upregulated by dMCAO but decreased following VX-765 treatment (*P* < 0.05). Moreover, VX-765 treatment reduced the number of TUNEL-positive cells and the occurrence of apoptosis in the ipsilateral hippocampus following dMCAO.

### VX-765 Protects Against Secondary Degeneration by Inhibiting the NF-κB and MAPK Pathways

**Fig. 6:**
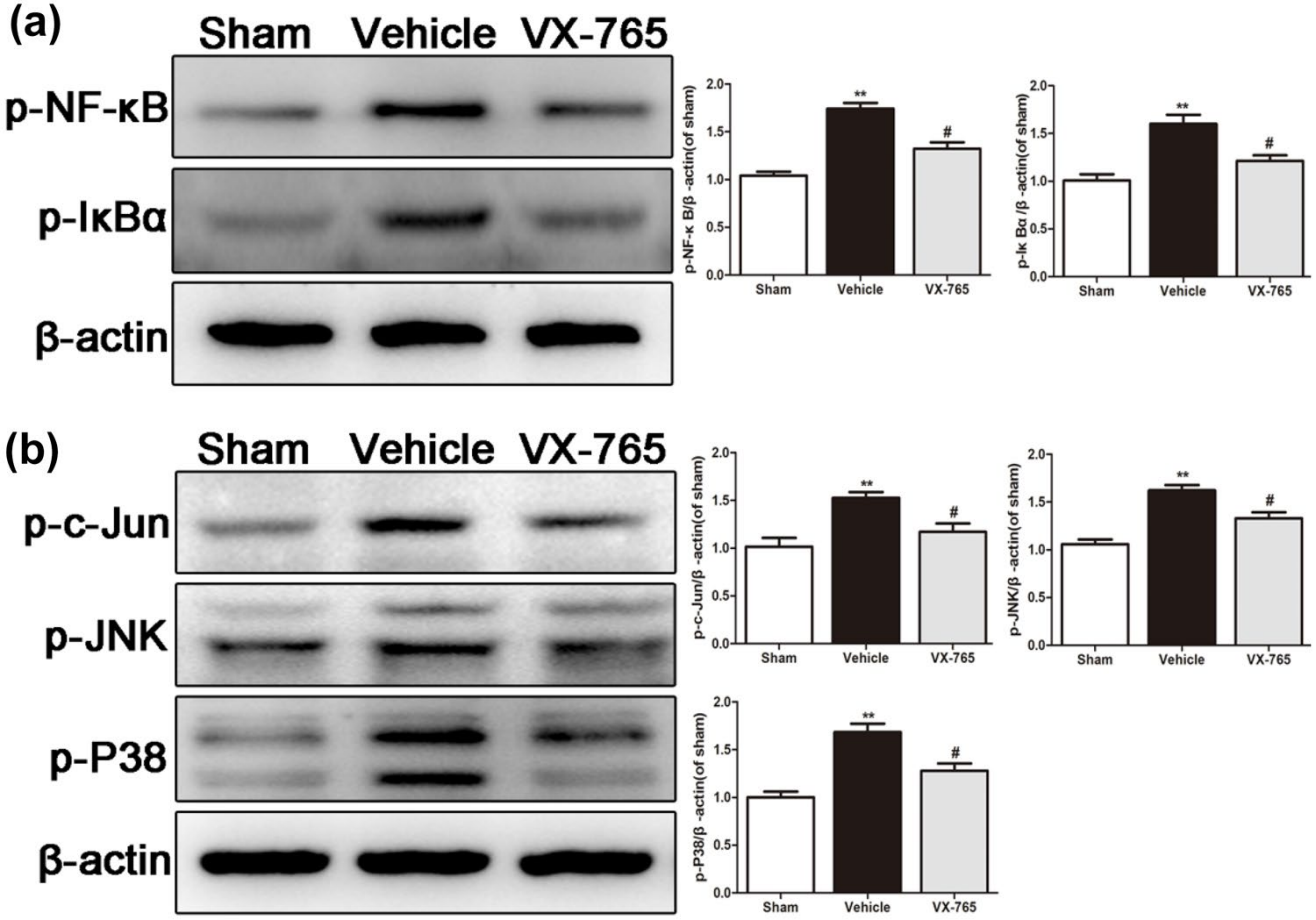
VX-765 attenuates secondary degeneration by inhibiting the NF-κB and MAPK pathways after dMCAO. **a** Western blot images indicating the expression of p-NF-κB and p-IκBα in the ipsilateral hippocampus after dMCAO. **b** Western blot images showing the expression of p–c-Jun, p-JNK, and p-p38 in the ipsilateral hippocampus after dMCAO, *n* = 6 per group. Data are shown as the mean ± SEM. ^**^P < 0.01 versus the sham group; ^#^*P* < 0.05 and ^##^*P* < 0.01 versus the vehicle group

The expression levels of proteins associated with the NF-κB and MAPK pathways were examined after considering the effects of classical NF-κB on the activation of glial cells and the inflammatory response after ischemia (Hayden and Ghosh 2008). The levels of p-NF-κB and p-IκBα were significantly higher in the ipsilateral hippocampus of the vehicle group than in the sham group. However, the expression level was significantly lower in the VX-765 group than in the vehicle group (*P* < 0.05, Fig. 6a). The levels of p–c-Jun, p-JNK, and p-p38 were higher in the ipsilateral hippocampus of the vehicle group than in the sham group but were significantly reduced following VX-765 treatment (*P* < 0.05, Fig. 6b). Therefore, these results revealed that VX-765 attenuated secondary degeneration by regulating the NF-κB and MAPK signaling pathways after dMCAO.

## Discussion

In this study, increased caspase-1 expression was associated with secondary degeneration, as evidenced by neuronal loss and glial activation in the ipsilateral hippocampus following focal cerebral ischemia. The caspase-1 inhibitor VX-765 suppressed the activation of caspase-1 and pyroptosis in the ipsilateral hippocampus after dMCAO. In addition, this was accompanied by downregulation of the expression of NLRP3, ASC, GSDMD, IL-1β, and IL-18. These effects are related to the reduction in secondary hippocampal degeneration and amelioration of cognitive decline after ischemic stroke. These results further revealed that secondary degeneration in the ipsilateral hippocampus may be related to poststroke cognitive decline and that VX-765 treatment reduced hippocampal Aβ deposition and neuronal synapse damage. This potentially ameliorated cognitive decline in focal cerebral ischemia in rats.

Caspase-1 (also known as IL-1β-converting enzyme) is an enzyme implicated in neuroinflammation, a crucial component of many diseases involving neurodegeneration (Mariathasan et al. 2004). Studies have shown that pharmacological inhibition of caspase-1 reduces ischemic neuronal damage and functional decline after cerebral ischemia (Li et al. 2019; Zhao et al. 2017). In addition, VX-765 has been reported to attenuate cognitive decline and brain inflammation and enhance synaptophysin protein levels in the AD mouse hippocampus (Flores et al. 2018). Several studies have shown that poststroke cognitive decline involves primary or secondary degeneration (Fernandez-Andujar et al. 2014; Xie et al. 2011). Secondary degeneration and inflammation have been observed in the ipsilateral hippocampus, as well as the activation of microglia and astrocytes after focal cerebral infarction (Chen et al. 2017). In this study, caspase-1 inhibition by VX-765 significantly improved cognitive impairment and secondary degeneration in the nonischemic hippocampus after focal cortical infarction. VX-765 treatment reduced pyroptosis activation in the nonischemic hippocampus, as evidenced by the decreased expression of NLRP3, GSDMD, ASC, IL-1β, and IL-18.

Aβ is the main component of senile plaques in the AD brain and is cytotoxic to neurons (Huang et al. 2018). In the distal MCAO model, abnormal Aβ accumulation contributes to secondary neurodegeneration in the ipsilateral thalamus (Groen et al. 2005). However, the mechanism of Aβ deposition in areas remote from the original ischemic lesions remains elusive. In this study, Aβ accumulation after focal cortical infarction was accompanied by neuronal loss and activation of astrocytes in the ipsilateral hippocampus. However, VX-765 treatment significantly reduced Aβ deposition and prevented secondary degeneration in the ipsilateral hippocampus after cerebral ischemia. This suggested that VX-765 has a protective effect on Aβ-related neurodegeneration. Consequently, VX-765 has been shown to attenuate brain inflammation and neuropathology by decreasing abnormal Aβ accumulation in AD mice (Flores et al. 2018). Since Aβ accumulation exerts neurotoxic effects via synaptic damage pathway (Ma et al. 2013), this study determined whether the reduction of synaptic damage is involved in the neuroprotective pathway of VX-765. BDNF plays an important role in the survival, differentiation, growth, and function of neurons (Corey et al. 2019). PSD95, Syna, and GAP43 are also considered neuronal plasticity markers because they promote synapse reconstruction and recovery (Mardones et al. 2019; Reddy et al. 2017). VX-765 treatment increased the expression levels of PSD95, Syna, and GAP43 in the ipsilateral hippocampus after dMCAO, which was accompanied by decreased Aβ accumulation. Aβ-induced neuronal death has been reported to be associated with caspase activation, Bax induction, and Bcl-2 downregulation (Selznick et al. 2000). In this study, Aβ deposition was associated with the downregulation of Bcl-2 and the upregulation of Bax and cC3 in the ipsilateral hippocampus after dMCAO. In addition, VX-765 treatment prevented Aβ expression and reduced neuronal apoptosis in the ipsilateral hippocampus after dMCAO. This suggested that VX-765 has a protective effect on the Aβ-induced apoptosis pathway.

The MAPK family comprises serine/threonine protein kinases, including the cellular signaling molecules p38 and JNK (Nithianandarajah-Jones et al. 2012). Activation of the MAPK family has been shown to increase stroke damage via the enhancement of neuronal apoptosis (Cui et al. 2007). Studies have shown that JNK accelerates stroke injury after activation, and the p38 expression signal can amplify the ischemia-induced inflammatory response (Kuan et al. 2003). However, inhibiting JNK and p38 expression ameliorates the prognosis outcomes following ischemic stroke. In addition, activated MAPKs contribute to IκB kinase activation, stimulate the release of the NF-κB dimer in the cytoplasmic NF-κB/IκB complex, and promote the nuclear translocation of NF-κB, causing an inflammatory response (Joo et al. 2007). We found that VX-765 reduced apoptosis and secondary degeneration in the ipsilateral hippocampus after focal cortical ischemia, mainly via the NF-κB and MAPK pathways.

## Conclusion

This study suggests that VX-765 treatment alleviates Aβ deposition, secondary degeneration, pyroptosis, and apoptosis in the ipsilateral hippocampus and ameliorates cognitive decline following focal cerebral ischemia. These effects may be mediated by the inhibition of MAPK and NF-κB pathway activation. Our findings provide evidence that the administration of a caspase-1 inhibitor can protect against secondary degeneration after ischemic stroke.

## Data Availability

The datasets generated during and/or analyzed during the current study are available from the corresponding author on reasonable request.
